# Exome sequencing and genome-wide copy number variant mapping reveal novel associations with sensorineural hereditary hearing loss

**DOI:** 10.1186/1471-2164-15-1155

**Published:** 2014-12-20

**Authors:** Rajini R Haraksingh, Fereshteh Jahanbani, Juan Rodriguez-Paris, Joel Gelernter, Kari C Nadeau, John S Oghalai, Iris Schrijver, Michael P Snyder

**Affiliations:** Department of Genetics, Stanford University School of Medicine, 300 Pasteur Dr., M-344A, Stanford, CA 94305 USA; Department of Pathology, Stanford University School of Medicine, Stanford, CA USA; Department of Psychiatry, Yale University School of Medicine, New Haven, CT USA; Department of Pediatrics, Stanford University School of Medicine, Stanford, CA USA; Department of Otolaryngology – Head and Neck Surgery, Stanford University School of Medicine, Stanford, CA USA; Stanford University School of Medicine, 3165 Porter Drive - Room 2270, Palo Alto, CA 94304 USA

**Keywords:** Hereditary Hearing Loss, *MYH7B*, Exome sequencing, Copy number variation, Array Comparative Genome Hybridization (aCGH)

## Abstract

**Background:**

The genetic diversity of loci and mutations underlying hereditary hearing loss is an active area of investigation. To identify loci associated with predominantly non-syndromic sensorineural hearing loss, we performed exome sequencing of families and of single probands, as well as copy number variation (CNV) mapping in a case–control cohort.

**Results:**

Analysis of three distinct families revealed several candidate loci in two families and a single strong candidate gene, *MYH7B*, for hearing loss in one family. *MYH7B* encodes a Type II myosin, consistent with a role for cytoskeletal proteins in hearing. High-resolution genome-wide CNV analysis of 150 cases and 157 controls revealed deletions in genes known to be involved in hearing (e.g. *GJB6*, *OTOA*, and *STRC*, encoding connexin 30, otoancorin, and stereocilin, respectively), supporting CNV contributions to hearing loss phenotypes. Additionally, a novel region on chromosome 16 containing part of the *PDXDC1* gene was found to be frequently deleted in hearing loss patients (OR = 3.91, 95% CI: 1.62-9.40, p = 1.45 × 10^-7^).

**Conclusions:**

We conclude that many known as well as novel loci and distinct types of mutations not typically tested in clinical settings can contribute to the etiology of hearing loss. Our study also demonstrates the challenges of exome sequencing and genome-wide CNV mapping for direct clinical application, and illustrates the need for functional and clinical follow-up as well as curated open-access databases.

**Electronic supplementary material:**

The online version of this article (doi:10.1186/1471-2164-15-1155) contains supplementary material, which is available to authorized users.

## Background

Hereditary sensorineural hearing loss (SNHL) is a highly prevalent disorder in humans, affecting 1 in 500 newborns[1]. There is considerable genetic heterogeneity underlying SNHL. Approximately 133 autosomal non-syndromic loci (55 dominant and 78 recessive) have been mapped, and within these, 78 genes are causally implicated in non-syndromic hearing loss: 30 for dominant and 48 for recessive hearing loss. In addition, there are three non-syndromic X-linked genes known to date (http://hereditaryhearingloss.org, accessed February 24, 2014). Despite the large number of implicated loci only one region has been shown to be a major etiological contributor to bilateral autosomal recessive non-syndromic hearing loss (ARNSHL). The *DFNB1A/B* locus contains the *GJB2* and *GJB6* genes, which encode the connexin 26 and connexin 30 proteins, respectively, and G*JB2* has been shown to be frequently mutated in individuals with severe ARNSHL[2, 3]. Because the region responsible for many of the remaining cases has not been identified there are likely to be other yet-to-be-discovered genetic contributors that may underlie a significant proportion of cases.

Most SNHL loci have been discovered using homozygosity mapping and other forms of linkage analysis in large consanguineous families [4] *.* There have been few genome-wide association studies on SNHL [5, 6], and most mutations associated with SNHL have been SNVs (http://deafnessvariationdatabase.com). Only one study has investigated the effects of copy number variants (CNVs) on SNHL in a comprehensive, though low-resolution fashion. This study found only one CNV, a deletion of the stereocilin gene *STRC,* that was associated with SNHL [6]. However, CNVs are enriched in genes involved in sensory perception of the environment [7] including smell and taste receptors [8]. Thus, there is a need to investigate the effects of CNV on SNHL in a high-resolution, unbiased, genome-wide manner, and further to investigate the integrated effects of multiple types of variants on this phenotype [9].

The advent of high-resolution, genome-wide variant mapping technologies, such as whole genome and exome sequencing, and microarray-based methods, now allows unbiased detection of the entire spectrum of genetic variants, including SNVs, indels, and CNVs, in individual genomes [10–14]. Exome sequencing studies have identified novel SNHL genes and/or mutations in probands, often followed by confirmation through limited analyses of other family members [9, 15–20].

In this study, we explored the utility of diverse and complementary high-resolution approaches to detect genetic variants associated with SNHL. We used multiple whole genome variant-mapping technologies, including exome sequencing and high-resolution array comparative genomic hybridization (aCGH), as well as familial and association strategies, to determine individually rare and frequent genetic contributors to SNHL in three families and in over 150 individual probands, for whom no conclusive genetic etiology had previously been established. We report the discovery of rare compound heterozygous mutations in the myosin heavy chain 7B gene, *MYH7B*, as a novel likely cause of SNHL, by exome sequencing a family of five individuals. We also report several individually rare, novel candidate mutations for SNHL, revealed by exome sequencing of two additional families (of four and five individuals, respectively) and 13 unrelated probands. Finally, we conducted the first high-resolution, genome-wide CNV investigation for hearing loss. We report several novel CNV associations found in a cohort of 150 affected individuals and 157 controls, including a deletion on chromosome 16 encompassing the *PDXDC1* gene, the has-mir-1972 micro RNA, and part of the *NPIP*. These results support our hypothesis that SNHL may manifest due to either underlying shared or individually rare genetic etiologies in different cases, and arise by multiple mechanisms.

## Results

### Two strategies to investigate novel genetic contributors to SNHL

We used two strategies to investigate the genetic variations underlying SNHL in individuals for whom no previous genetic etiology had been established. The first strategy involved analyzing novel SNV and indel associations with SNHL using exome sequencing in three affected families and 13 additional isolated probands (Table 1). The second approach involved analyzing genome-wide copy number changes using high-resolution aCGH to discover CNVs associated with SNHL in a cohort of 150 probands and 157 controls (Table 2). This cohort includes the 13 isolated probands from the exome sequencing study. This multiple strategy approach was designed to provide a detailed, yet comprehensive investigation of the type and nature of mutations affecting hearing.

**Table 1:** **Exome sequencing study subjects**

Study number	Sex	Ethnicity	Age of onset	Level and type of hearing loss	Condition	Family history	Other findings	Sequence variation
1	M	Mexican	Congenital	Bilateral profound, sensorineural	Stable	Similarly affected sibling	None	*GJB2*, *SLC26A4*: negative
2	F	Native American	Congenital	Bilateral moderate, sensorineural	Unknown	Negative	None	*GJB2*: negative
3	F	East Asian	Age 9	High frequency sensorineural hearing loss, left more severe	Unknown	Negative	None	*GJB2* and *GJB6*: negative; *SLC26A4*: heterozygous for c.463 A > G, p.Met155Val (a novel variant of uncertain pathogenic significance)
4	M	Mexican	Congenital	Bilateral profound, sensorineural	Stable	Similarly affected sibling	None	*GJB2* and *GJB6*: negative
5	M	Caucasian	Age 2	Bilateral mild to moderate, sensorineural	Progressive	Mother with bilateral severe hearing loss, recognized around age 16	None	*GJB2*: negative
6	F	Caucasian	Congenital	L - profound, R - moderate, sensorineural	Stable	Similarly affected sibling	None	*GJB2*: negative
7	M	Mexican	Congenital	Bilateral severe to profound, sensorineural	Stable	Negative	None	*GJB2*: negative
8	F	Caucasian-East Asian	Age 1	L- severe, R - profound, sensorineural	Unknown	Unknown	None	*GJB2*: negative
9	M	Caucasian-African American	Congenital	L - moderate, mixed hearing loss, R - moderate, sensorineural	Stable	Negative	None	*GJB2*: negative
10	M	Mexican	Congenital	Bilateral moderate, sensorineural	Stable	Hearing of unknown etiology loss on paternal side	None	*GJB2*, *SLC26A4*: negative
11	F	Mexican	Congenital	Bilateral moderate, sensorineural	Stable	Negative	None	*GJB2*: negative
12	M	Caucasian	Congenital	Bilateral moderate, sensorineural	Stable	Negative	None	*GJB2*: negative
13	M	East Asian	After age 7, confirmed at age 14	Bilateral profound, sensorineural	Progressive	Negative	None	*GJB2*: heterozygous for c.11G > A, p.Gly4Asp (a variant of uncertain pathogenic significance)
F1.1	M	Middle Eastern	N/A	N/A	Unaffected	Affected offspring	None; consanguineous	
F1.2	F	Middle Eastern	N/A	N/A	Unaffected	Affected offspring	None; consanguineous	
F1.3	M	Middle Eastern	Congenital	Bilateral severe to profound, sensorineural	Progressive	Proband with similarly affected siblings	Megalocornea with secondary glaucoma	*GJB2* and *GJB6*: negative
F1.4	M	Middle Eastern	Congenital	Bilateral severe to profound, sensorineural	Progressive	Similarly affected siblings	Megalocornea with secondary glaucoma	
F1.5	F	Middle Eastern	Congenital	Bilateral severe to profound, sensorineural	Progressive	Similarly affected siblings	Megalocornea with secondary glaucoma	
F2.1	M	Caucasian	N/A	N/A	Unaffected	Affected offspring	None, normal chromosomes	
F2.2	F	Caucasian	N/A	N/A	Unaffected	Affected offspring	None, normal chromosomes	
F2.3	F	Caucasian	Congenital	Bilateral moderate, sensorineural	Stable	Proband with similarly affected identical twin	None	*GJB2*, *GJB6*, mitochondrial mutation panel: negative
F2.4	F	Caucasian	Congenital	Bilateral moderate, sensorineural	Stable	Similarly affected identical twin	None	*GJB2*, *GJB6*, mitochondrial mutation panel: negative
F2.5	M	Caucasian	Congenital	Bilateral moderate, sensorineural	Stable	Affected sibling	Multiple congenital abnormalities partial chromosome 7 deletion	*GJB2*, *GJB6*, mitochondrial mutation panel: negative
F3.1	M	Caucasian	N/A	N/A	Unaffected	Affected offspring	None	
F3.2	F	Caucasian	Unknown	Mild	Unknown	Affected offspring, affected mother	Mother reported to have a white forelock	
F3.3	M	Caucasian	Failed initial newborn screening but passed a rescreen. At age 3 mild to moderate hearing loss was identified	Bilateral mild to moderate	Progressive	Proband with similarly affected sibling, mildly affected mother and maternal grandmother	None	*GJB2, GJB6, SLC26A4*: negative
F3.4	M	Caucasian	Congenital	Bilateral mild to moderate	Progressive	Similarly affected sibling, mildly affected mother and maternal grandmother	None	

*Abbreviations:* F – female, M – male. Ethnicity was determined using Principal Components Analysis with the Human Genome Diversity Panel.

**Table 2:** **CNV association study samples**

Phenotype	Total	Gender		Ethnicity							
		Female	Male	Caucasian	Mexican	East Asian	Native American	African American	African American-Caucasian	Caucasian-East Asian	Middle Eastern
**Affected**	**150**	**65**	**85**	**44**	**59**	**27**	**6**	**4**	**6**	**4**	**0**
**Unaffected**	**157**	**79**	**78**	**132**	**16**	**5**	**0**	**2**	**0**	**2**	**1**

Affected individuals were recruited at Stanford University under IRB approval. Unaffected individuals matched for sex, age, and ethnicity were collected under IRB approval at Stanford University (n = 31), Mount Sinai University (n = 88) and Yale University (n = 38) using identical selection criteria. Ethnicity was determined using Principal Components Analysis with the Human Genome Diversity Panel.

### Exome sequencing of individuals with familial and sporadic hearing loss

**Figure 1:**
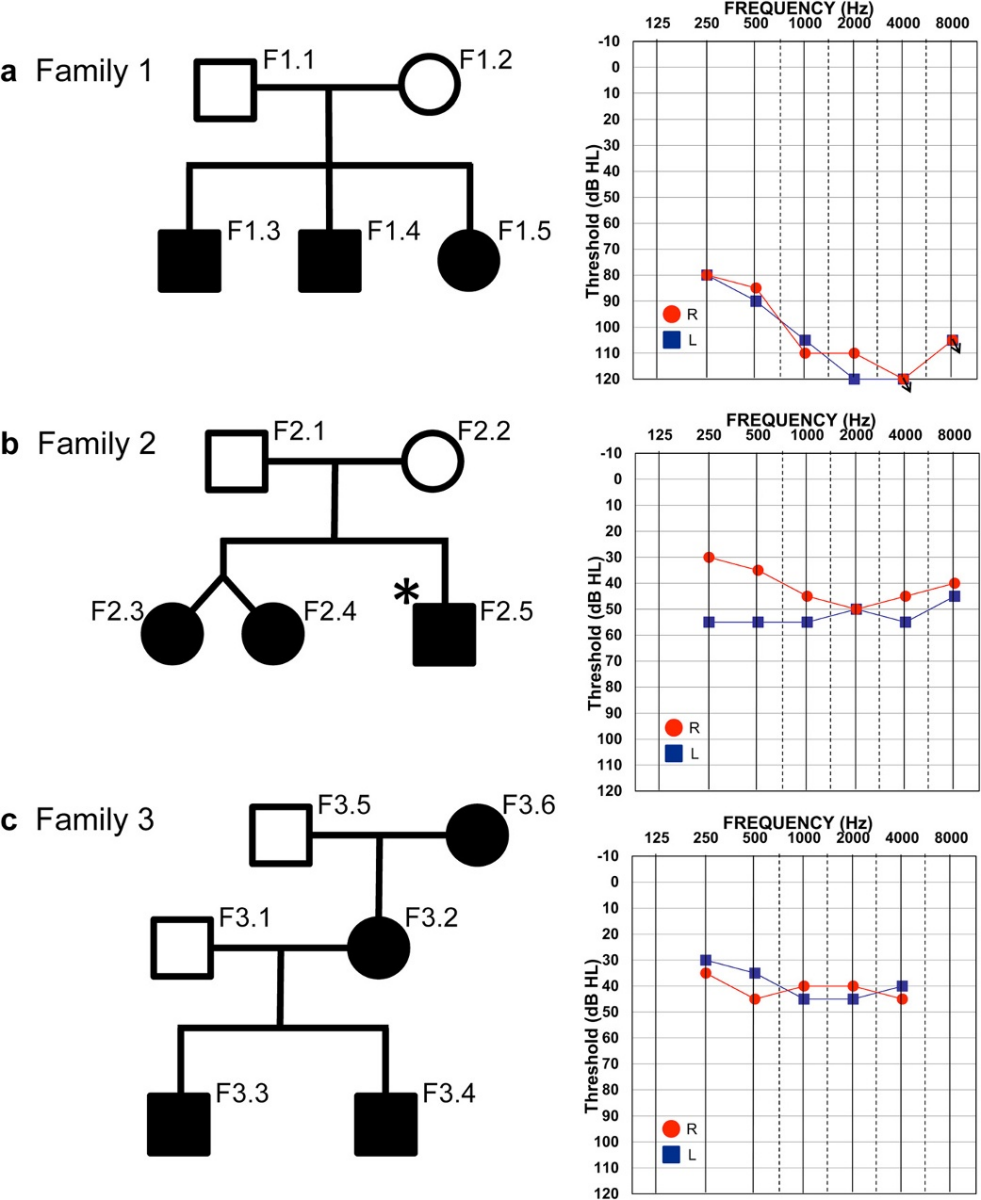
**Families affected with syndromic hereditary hearing loss.** Filled symbols indicate individuals affected with hearing loss. Audiograms are representative of the hearing loss in all affected members of each respective family. **a**. Family 1 showing Mendelian recessive inheritance of severe to profound hearing loss. The audiogram corresponds to proband F1.5. **b**. Family 2 showing Mendelian recessive inheritance of moderate hearing loss or dominant de novo inheritance in the twins. The audiogram corresponds to proband F2.4. **c**. Family 3 showing Mendelian dominant inheritance of mild hearing loss. The audiogram corresponds to proband F3.3.

**Figure 2:**
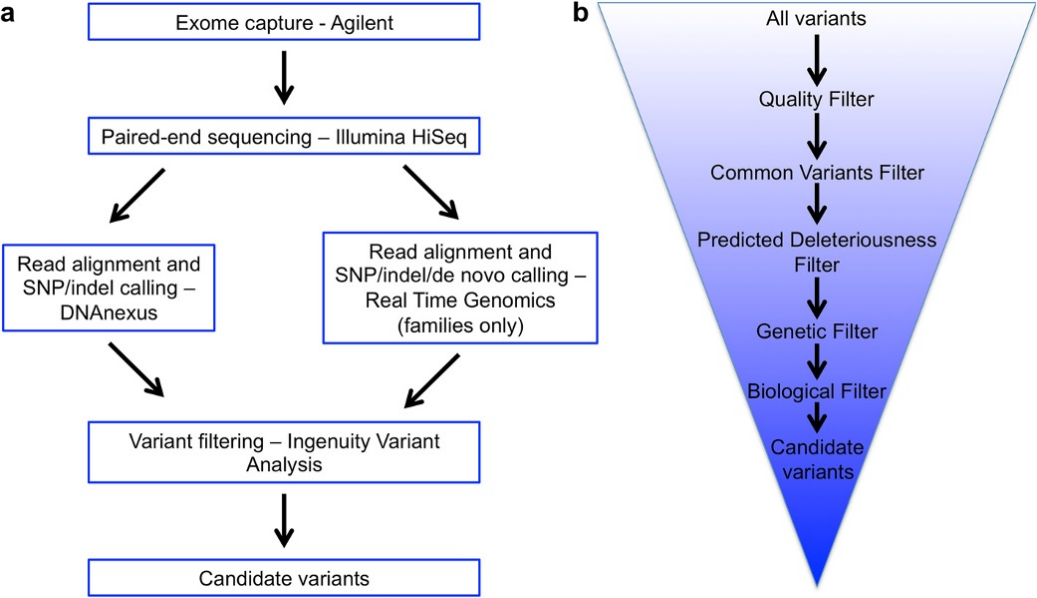
**Exome sequencing analysis and variant filtering scheme. a**. Analysis protocol for exome sequencing study. **b**. Variant filtering scheme using Ingenuity Variant Analysis (IVA) for variant prioritization of exome data.

We performed exome sequencing on three families with different levels of sensorineural hearing loss. The severity of the hearing loss was determined by behavioral pure tone audiometry. Family 1 is of middle-eastern descent and afflicted with severe-profound bilateral hearing loss (>90 dB) and megalocornea with secondary glaucoma. Family 2 is of European-Caucasian descent and afflicted with moderate hearing loss (~50 dB). Family 3 of European-Caucasian descent has mild hearing loss (~40 dB) (Figure 1). We also performed exome sequencing on 13 additional probands and searched for rare, highly penetrant SNVs and indels that may explain the phenotype. In each case we aligned 100 bp paired-end sequencing reads and called SNVs and indels using the Nucleotide-level Variation tool from DNAnexus. For the three families we also independently aligned reads and called SNVs and indels using the Variant 1.0 algorithm from Real Time Genomics (RTG) (Figure 2a). We note that the total number of variants called per genome differ significantly between the two algorithms due to technical differences. DNAnexus calls variants individually in each genome and then we used family structure to apply various segregation models. RTG uses the familial structure a priori to call variants segregating in the family under various inheritance models (see Methods). We then used Ingenuity Variant Analysis (IVA) to filter the variants based on quality, frequency in known populations, predicted deleteriousness, genetic analysis (families only) and biological context (Figure 2b). We discovered multiple potential genetic etiologies in the studied families and in the individual probands.

#### Compound heterozygous missense mutations in MYH7B are the likely cause of hearing loss in family 1

Family 1 has a severe hearing defect that segregates in a recessive manner (Figure 1a). The exomes of the two unaffected parents and three affected children were analyzed as described above. Of the 88,975 and 346,430 variants called by RTG and DNAnexus, respectively, (spanning 16,928 and 18,811 genes, each), 78,579 and 276,015, respectively, had a call quality of at least 20 in all samples. Variants with an allele frequency of 3% or greater in the genomes of the 1000 genomes project, the public Complete Genomics genomes, or the NHLBI ESP exomes (collectively referred to as public genomes hereafter), were excluded, leaving 17,249 variants for RTG and 85,007 for DNAnexus. 6,312 and 9,618 of the RTG and DNAnexus variants, respectively, were experimentally observed or predicted to be damaging by IVA. Further filtering based on segregation in a recessive fashion yielded six SNP/indel (RTG) and three SNP (DNAnexus) variants. These independent analyses had only two variants in common. They were both heterozygous missense mutations in the *MYH7B* gene on chromosome 20q11.22. Relaxing the rarity filter to encompass variants that occurred at a frequency ≤ 15% in the public genomes did not yield any additional candidates.

**Figure 3:**
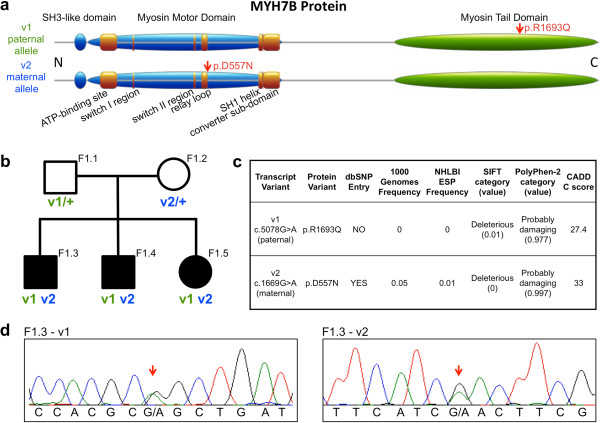
**Compound heterozygous mutations in the** ***MYH7B*** **gene segregate with the hearing loss in Family 1. a**. MYH7B protein showing functional domains and locations of missense mutations in the paternal and maternal alleles. **b**. Segregation of heterozygous missense mutations in the *MYH7B* gene in Family 1. **c**. Description and population frequencies of rare paternal and maternal alleles of *MYH7B*. **d**. Verification of compound heterozygous mutations in proband F1.3 by Sanger sequencing. These data are representative of those of the other family members.

One of the two *MYH7B* variants (v1: p.Arg1693Gln) is heterozygous in the father and the other (v2: p.Asp557Asn) is heterozygous in the mother (Figure 3a). Each parent carries only one mutant allele but all three affected children are compound heterozygous for both mutations (Figure 3b). The maternal variant was present in dbSNP (Build 137) and present in the 1000 Genomes Project and Exome Sequencing Project samples at a frequency of 0.05% and 0.01%, respectively. The paternal variant was not present in dbSNP, the 1000 Genomes Project, or the Exome Sequencing Project samples. Neither variant was present in any of the other exomes of probands in our cohort. Thus, both variants appear to be rare and the paternal variant may be private to this family. We further analyzed these mutations using the Combined Annotation Dependent Depletion algorithm (CADD), a general framework for estimating the relative pathogenicity of genetic variants in humans [21]. They received scaled C scores of 27 and 33 for v1 and v2, respectively, indicating that these variants are in the 0.2 and 0.05 percentile of most deleterious substitutions in the human genome, respectively (Figure 3c). These variants were verified by Sanger sequencing in all family members (Figure 3d).

*MYH7B* encodes a heavy chain of myosin II, a member of the motor-domain superfamily. The myosin II molecule is a multi-subunit complex made up of two heavy chains and four light chains. The heavy chain comprises a catalytic globular motor domain, which carries out ATP hydrolysis and interacts with actin, and a tail domain in which heptad repeat sequences promote dimerization by interacting to form a rod-like alpha-helical coiled coil. The maternal variant lies in the relay loop of the catalytic motor domain and the paternal variant is located in the tail domain, which is responsible for dimerization (Figure 3a). The *MYH7B* gene has not been previously implicated in hearing loss but has been linked to differentiation of inner ear hair cells [22] and shown to control actin networks within neurons [23].

#### MYH7B is expressed in the inner ear

We next examined expression of the *MYH7B* gene in the literature and in the Allen Brain Atlas. *MYH7B* was found to be concordantly expressed in embryonic mouse inner ear tissue but not in non-inner ear tissue, with atonal homolog 1a, *ATOH1*, a gene required for hair cell differentiation [22]. This concordant expression likely indicates a role for *MYH7B* in development of hair cells in the inner ear. In addition, microarray data from the Allen Brain Atlas indicates high *MYH7B* expression in the primary auditory cortex and regions of the auditory pathway such as the cochlear nuclei and inferior colliculus, in adult humans [24]. Furthermore, a reduction in *MYH7B* expression in cultured mature rat hippocampal neurons can cause profound alterations to dendritic spine morphology, excitatory synaptic strength, and the actin cytoskeleton [23]. It is possible that these effects may extend to other neuronal tissues including the auditory complex.

#### Variant filtering results in a shortlist of potential causative mutations underlying the hearing loss in families 2 and 3

The hearing loss in family 2 is moderate and bilateral and appears to segregate in either a recessive or dominant de novo fashion in female twin offspring (Figure 1b). An additional male sibling has multiple congenital abnormalities, including hearing loss, which can be explained by chromosomal abnormalities that are absent in the twins. Accordingly, his hearing loss is different from that in the twins as confirmed by audiogram (not shown) and he was excluded from the analysis of this family. The exomes of the other four family members were analyzed as above. In this family, 469,864 variants were called in total, either by RTG (94,974 total), DNAnexus (438,031 total), or both (63,141). Filtering was performed using IVA on the union set of variants called by both algorithms to maximize findings. After removing common variants and low quality calls, two genetic models were applied; dominant de novo and recessive inheritance. We searched for dominant de novo mutations that were called by both RTG and DNAnexus that occurred in both twins. No such mutations were found. However, two potential candidates were found by DNAnexus only. These were a heterozygous in-frame deletion, (p.Ala23-Leu25del), in the *CTBS* gene (encoding Di-N-acetylchitobiase), and a heterozygous missense mutation (p.Thr26Ala) in the *RBMXL1* gene (encoding RNA binding motif protein, X-linked-like 1) and in an intron of the gene *CCBL2* (encoding Cysteine Conjugate-Beta Lyase 2). The search for underlying variants following a recessive pattern of inheritance did not generate any robust candidates.

Family 3 is characterized by mild to moderate bilateral hearing loss appearing to segregate in an autosomal dominant fashion (Figure 1c). However, the level of hearing loss in the mother is milder than that of the children. This indicates either incomplete penetrance of the causative allele that is segregating in an autosomal dominant manner, or compounding of the phenotype due to the additive effects of some putative paternal variant along with the maternal variant. It is also possible that the hearing loss in the mother is different from that in the children. However, this scenario is less likely given the seemingly Mendelian inheritance.

The exomes of all four family members were analyzed as above. In total, 327,843 variants were called either by RTG (91,397 total), DNAnexus (293,696 total), or both (57,250). Filtering was performed in IVA on the union set of variants called by both algorithms. After removing common variants and low quality calls, only 14 variants were called by both RTG and DNAnexus, were predicted to be deleterious by IVA, and segregated in an autosomal dominant manner (Table 3). All but one of these has been reported in dbSNP. In this set, seven missense mutations were found, of which four were predicted to be damaging and three were predicted to be activating by SIFT. Five of these were heterozygous in all affected individuals while two were homozygous in the affected mother and heterozygous in the children. Of these seven variants, six occur in biological pathways at most two nodes away from some gene known to be involved in autosomal dominant non-syndromic hearing loss. These six missense variants located in networks containing known hearing loss genes (missense variants in Table 3 except the *GAL3ST2* mutation) are the present leading causative candidate mutations in this family.

**Table 3:** **Candidate variants for Family 3**

Chr	Position (hg19)	Reference Allele	Sample Allele	Gene region	Gene symbol	Transcript variant	Protein variant	Genotype			Translation Impact	dbSNP ID (Build 137)	1000 genomes frequency (v3)
								F3.2	F3.3	F3.4			
1	908929	C	T	Exonic	PLEKHN1	c.1238C > T; c.1343C > T	p.S413L; p.S448L	het	het	het	missense	142080242	0.27
1	3440753	G	A	Exonic	MEGF6	c.539C > T	p.P180L	het	het	het	missense	41307039	0.51
1	15546259	G	A	3'UTR	TMEM51	c.*20G > A		het	het	het		199958353	
1	68649258	C	T	Intronic; microRNA	WLS; mir-1262	c.373 + 10380G > A; c.107-24328G > A; c.379 + 10380G > A		het	het	het		147113488	0.65
1	234742618	G	A	3'UTR	IRF2BP2	c.*265C > T		het	het	het		192276553	0.33
1	245772651	C	G	Exonic	KIF26B	c.1735C > G	p.L579V	hom	het	het	missense	61754955	2.5
2	68546588	C	A	5'UTR	CNRIP1	c.-56G > T		het	het	het		185949133	2.35
2	86439256	A	T	Intronic; 3'UTR	MRPL35	c.513-293A > T; c.*1465A > T		het	het	het		192903152	0.14
2	97531651	G	A	Exonic	SEMA4C	c.274C > T	p.P92S	het	het	het	missense	141610691	
2	99978074	A	T	Exonic	EIF5B	c.710A > T	p.E237V	het	het	het	missense	201583340	
2	109087823	AAA		Exonic; ncRNA	GCC2	c.2038_2040delAAA	p.680delK	het	het	het	in-frame		
2	111875388	C	T	Exonic	ACOXL	c.1738C > T	p.L580F	hom	het	het	missense	193151657	0.7
2	233630577	G	A	3'UTR; Intronic	GIGYF2; KCNJ13	c.*2324C > T; c.532 + 4431G > A; c.*2886C > T		het	het	het		74547374	0.65
2	242742842	A	G	Exonic	GAL3ST2	c.458A > G	p.Y153C	het	het	het	missense	139344622	0.05

Family 3 variants with quality >20, frequency in the public genomes < 3%, called by both RTG and IVA as predicted deleterious, and showing dominant inheritance patterns. The chromosomal locations, gene regions, gene symbols, transcript variants, protein variants, genotype, translation impact, dbSNP identifier, and 1000 Genomes Project frequency are shown.

*Abbreviations:* het – heterozygous, homo – homozygous.

#### Exome sequencing of individual probands reveals rare deleterious mutations in genes known to be associated with hearing loss

**Figure 4:**
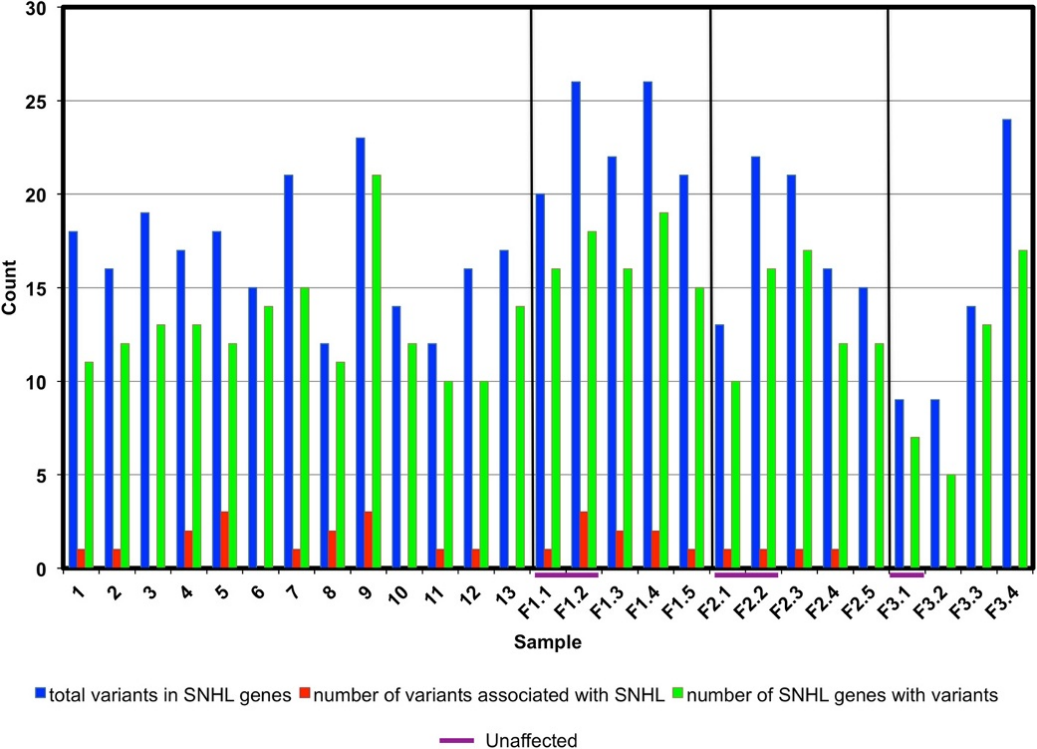
**Predicted deleterious variant load in known SNHL genes derived from exome sequencing of SNHL probands.** Black lines demarcate families. Purple bars indicate unaffected parents of probands in families. All included variants had a call quality greater than 20, and a frequency less than or equal to 15% in the 1000 genomes project, ESP, and Complete Genomics. The data represents a total of 134 variants in 46 genes (exons, splice sites, and miRNAs only).

It is unknown how often sequencing of individuals with hearing loss will identify likely underlying causes of the disease. We therefore also sequenced the exomes of 13 individual probands for which additional family members were not available. We used DNAnexus Nucleotide-Level Variation Analysis to detect SNPs and indels in each proband. Between 114,135 and 228,298 variants were found in each exome (mean = 188,659 variants per exome). Using the IVA variant filtering scheme described in the methods and published at https://variants.ingenuity.com/Haraksingh-etal-2013-HHLa, 21,554 potentially deleterious variants in 9,715 genes were revealed in this set of probands. Of these 133 variants occurred in 46 genes that have previously been associated with hearing loss. Between 12–23 predicted deleterious variants (mean = 17) located in 10–21 genes (mean = 13) known to be associated with hearing loss were found in each proband (Figure 4). In each proband, between one and six known hearing loss genes (mean = 3) with more than one predicted deleterious mutation were found.

In five of the 13 probands likely causative mutations can be identified. These are rare homozygous variants that are predicted to be damaging in known hearing loss genes. Proband 2 carries a stop loss in the *MYO7A* gene (p.*1179Gly) and a missense mutation (p.Pro426Leu) in the *MYO1A* gene. Proband 3 has a missense mutation (p.Leu2886Phe) in the *USH2A* gene that has been previously associated with Usher Syndrome in a Spanish family. There is a rare in-frame variant (p.398delGln) in the *TRIOBP* gene in probands 7 and 13, and proband 13 carries a missense mutation (p.Met6159Val) in the *GPR98* gene. Proband 9 carries a missense mutation (p.Lys130Glu) in the *USH1G* gene. These damaging homozygous mutations are the strongest candidates for causing hearing loss in the five associated probands. Retinal abnormalities had not been observed in the two probands with mutations in Usher syndrome genes, but both were young children.

Seven of the remaining eight probands carry at least one rare homozygous variant in genes one node away from a known hearing loss gene in Ingenuity-curated biological pathways. These variants were not found in the unaffected family members from our cohort. These rare, homozygous, deleterious variants represent the most likely causative alleles in these probands. The variants can be viewed at https://variants.ingenuity.com/Haraksingh-etal-2013-HHLb by invoking the ‘Homozygous’ filter followed by the ‘Hearing-relevant’ filter after the ‘Rarity’ filter.

Additionally, many genes were found containing recurrent predicted deleterious variants in at least two probands of our cohort. For example, under a dominant model, 398 genes contained recurrent variants in more than two probands and not in the unaffected members of families 1, 2, or 3. These genes may represent additional recurrent candidates underlying the hearing loss in our set of probands. The results of this analysis and other adjustments to the genetic model can be explored at https://variants.ingenuity.com/Haraksingh-etal-2013-HHLb. The dominant analysis results are obtained by moving the ‘Homozygous’ and ‘Hearing-relevant’ filters to the bottom of the cascade.

Overall, these results indicate that strong candidates can often be found by exome sequencing of genomic DNA of hearing loss patients. In other cases, larger numbers of candidates can be identified, the meaning of which is more difficult to distil.

### Genome-wide CNV mapping reveals several CNVs associated with SNHL

In our second approach, we carried out a high-resolution, genome-wide CNV association study of SNHL using 150 affected individuals, including the 13 isolated probands whose exomes were sequenced, and 157 controls. We mapped CNVs by aCGH on the NimbleGen 2.1 M CNV array, the most sensitive array-based CNV detection platform available at the time [13]. We then called CNVs using two algorithms, Nexus Copy Number 6 (Biodiscovery) and NimbleScan 2.6 (NimbleGen). Association testing was performed for genomic regions affected by CNVs including single loci, genes, and pathways, as well as for overall CNV load. We found an associated deletion on chromosome 16 encompassing the *PDXDC1* gene (OR = 3.91, 95% CI: 1.62-9.40, p = 1.45 × 10^-7^) as well as other less significant CNV associations. Additionally, we performed SNP genotyping of 150 cases and 28 controls using the Illumina 1 M SNP array and carried out a genome-wide association study (GWAS) using our cases and a large set of publicly available controls. Finally, we investigated whether there were combined effects of SNV and indel mutations with CNVs in the same locus for the 13 individual probands. The latter two approaches did not yield significant results.

In total, 155,634 CNVs were called by Nexus (12,555 total, 6,282 unique in cases and 143,079 total, 93,446 unique in controls), and 310,753 CNVs were called by NimbleScan (146,223 total, 34,939 unique in cases and 164,530 total, 37,202 unique in controls). We define a unique CNV call as one that contains a unique pair of start and end coordinates. 1,726 and 20,510 unique CNVs were common to both the case and control groups as called by Nexus and NimbleScan respectively.

Between two and 6,172 CNVs were called by Nexus (median = 89), and between 394 and 1,731 were called by NimbleScan (median = 1,036.5) in the individual genomes (Additional file 1: Figure S1). It was found that the individual cases and controls have similar genome-wide CNV loads. (However, note that a handful of outliers in the control group showed hundreds more Nexus CNV calls than the rest of the cohort). The Nexus CNV calls tend to be much larger for the cases than the controls. There is an enrichment of CNV calls between 30–80 kb in the cases, as well as a higher relative frequency of Nexus CNV calls that are greater than 100 kb. The NimbleScan case and control CNV calls are generally similar in size (Additional file 2: Figure S2).

**Figure 5:**
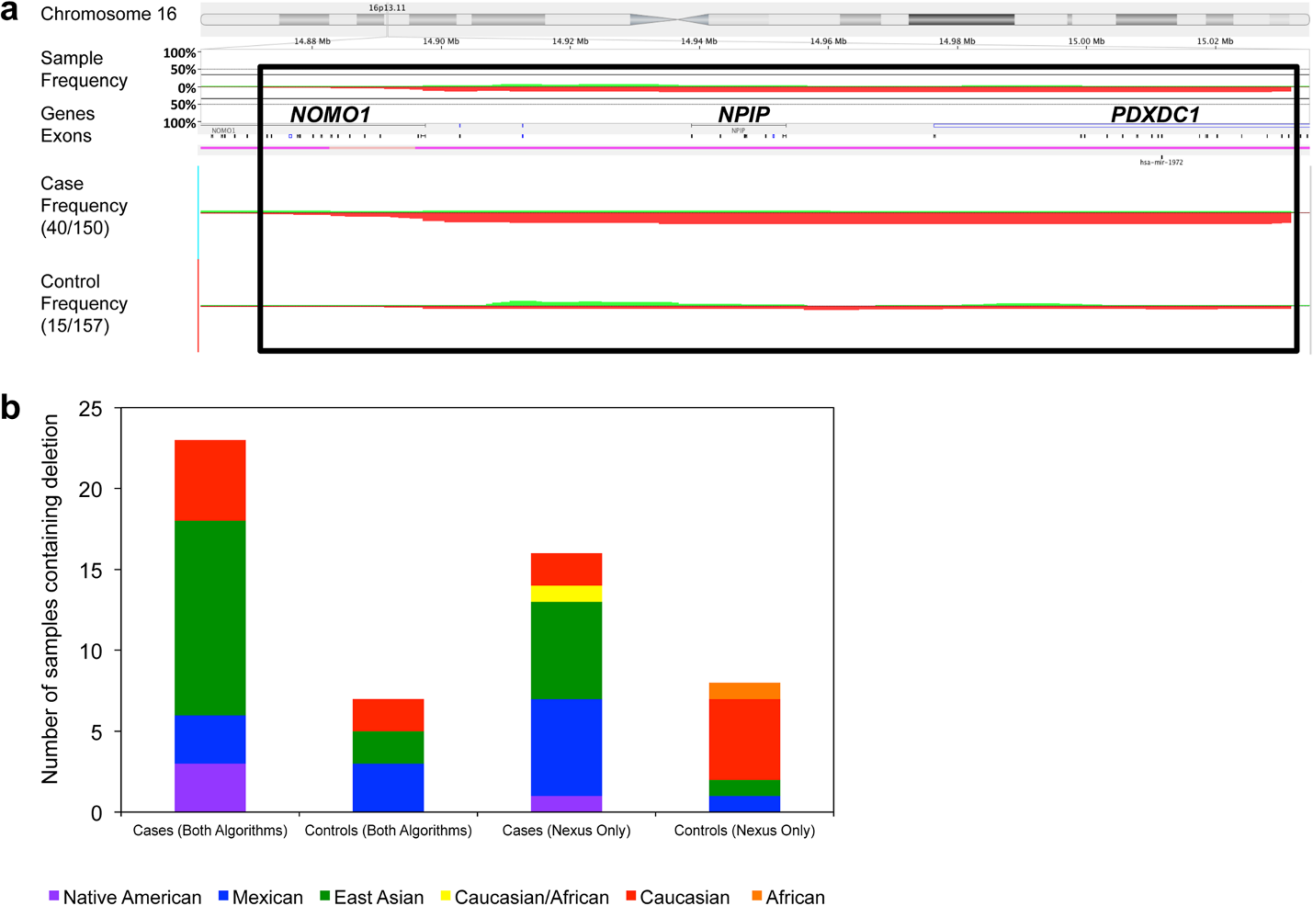
**Chromosome 16p13.11 deletion associated with SNHL. a**. The frequencies of the deletion on chromosome 16 encompassing part of the *PDXDC1* gene are indicated for the entire sample set, as well as separated out for the affected individuals and controls (highlighted in the black box). Green shading indicates duplications whereas red shading indicates deletions. The horizontal tracks indicate the coordinates of the position along chromosome 16p13.11 (hg18). Gene and exon tracks are included. **b**. The frequencies of the deletion among the various ethnic groups; African, Caucasian, East Asian, Mexican, and Native American. Frequencies were calculated separately for CNVs called by both algorithms and for CNVs called by one algorithm (Nexus) only.

CNVs were tested for association with the phenotype using the *Classic* calculation option of the *Comparisons* function in Nexus 6.0. Smallest regions of overlap of the individual CNVs were used. The most significant association is an approximately 72.5 kb (smallest region of overlap) deletion on chromosome 16 (hg18; chr16:14,956,245-15,028,783) encompassing the first 15 exons of the *PDXDC1* gene, the has-mir-1972 micro RNA, and the intergenic region between *PDXDC1* and the upstream *NPIP* gene (Figure 5a). Some of the individual deletions extend far enough upstream to include the *NPIP* gene as well as the five 3’ most exons of the *NOMO1* gene which is further upstream. This region was previously reported in the Database of Genomic Variants (DGV) and is thought to be the result of a duplication expansion in the human genome. The smallest region of overlap of the deletions is called in 23 cases and 7 controls by both algorithms independently producing an odds ratio of 3.91 (95% CI: 1.62-9.40, p = 1.45 × 10^-7^). An additional 17 subjects and eight controls carry the deletion as called by a single algorithm. Counting CNVs called by at least one algorithm, indicates that the deletion is present in 40 affected individuals and 15 controls producing an odds ratio of 3.47 (95% CI: 1.82-6.60). The deletion is present in the same number of Mexican cases and controls but significantly more East Asian (12 versus two) and Caucasian (five versus two) cases than controls when considering cases where both algorithms called the CNV (Figure 5b).

We observed deletions encompassing the entire *STRC* gene in seven cases and two controls as called by both algorithms, and in an additional control called by just the NimbleScan algorithm. The deletions ranged in size from 70–239 kb with the smallest region of overlap being hg18; chr15:41,639,153-41,709,787. This is the only CNV that has previously been reported to be associated with mild to moderate hearing impairment in *GJB2* mutation negative probands [6].

Each gene in the NCBI Reference Sequence Database (RefSeq), including 10 kb up and downstream of the gene, was tested for association with the phenotype under the premise that different mutations in the same gene can lead to the phenotype. The frequency at which each gene overlapped a CNV by at least one base pair was calculated for the cases and controls. A Fisher’s Exact Test was performed to determine whether the frequency differences between the cases and controls were significant. Associations that either contained the lowest p-values (before Bonferroni correction, which is frequently too stringent for GWAS studies) with the case frequency being higher than the control frequency, the lowest control frequency, or the most consistent trend from both CNV calling algorithms were the most functionally promising (Table 4). As expected, the three genes in the deletion on chromosome 16 (*NOMO1*, *NPIP* and *PDXDC1)* found to be associated were among the top candidates in this second set of association tests. Additionally, the *OTOA* gene, known to be associated with SNHL [1], was found to be significantly associated in this cohort. The *NBPF4* gene was found to be associated as well. This gene has no known function, but it is one of five genes that lies within the region of overlap of two previously discovered deafness associated genomic regions, *DFNB82* and *DFNB32* [15].

**Table 4:** **Interesting genes significantly associated with SNHL**

Gene	Nexus			NimbleScan		
	Case frequency	Control frequency	OR 95% CI FET p-value	Case frequency	Control frequency	OR 95% CI FET p-value
*NOMO1*	41	13	4.20	9	4	2.46
			2.14-8.21			0.74-8.16
			1.88E-05			1.63E-01
*GRAPL*	68	38	2.62	43	27	1.95
			1.61-4.26			1.13-3.36
			1.26E-04			2.09E-02
*PDXDC1*	41	16	3.33	24	9	3.15
			1.78-6.26			1.41-7.03
			1.27E-04			5.17E-03
*FCGR2C*	45	23	2.51	14	9	1.70
			1.43-4.42			0.71-4.06
			1.54E-03			2.81E-01
*NBPF4*	18	6	3.45	1	1	1.05
			1.33-8.96			0.07-17.00
			9.96E-03			1.00E + 00
*NPIP*	44	28	1.92	41	17	3.12
			1.12-3.30			1.68-5.79
			2.21E-02			2.56E-04
*FAM115A*	45	29	1.91	23	16	1.6
			1.12-3.25			0.81-3.18
			2.34E-02			2.30E-01
*OTOA*	18	8	2.56	5	1	5.13
			1.08-6.07			
						0.59-44.47
			3.97E-02			1.15E-01

Functionally interesting genes containing CNVs showing statistically significant associations with SNHL. The genes, case and control frequencies using both CNV calling algorithms, odds ratios, 95% confidence intervals, and p-values derived from Fisher’s Exact Tests are listed.

In order to test the hypothesis that different individuals may carry distinct mutations in a particular pathway which all result in the same phenotype, we carried out pathway association tests. Each pathway in the Kyoto Encyclopedia of Genes and Genomes (KEGG) containing a gene previously known to be associated with hearing loss was tested for association in this cohort. A Fisher’s Exact Test was used to determine if particular pathways were significantly enriched for CNVs in the cases versus the controls. No such pathway was found.

Finally, the CNV load in the cases versus controls of the set of 46 genes known to be associated with SNHL was tested. There was no significant difference in CNV load in the cases versus the controls for this set of genes.

#### Combined effects of CNVs and point mutations in the DFNB1 locus may explain the hearing loss in several probands

Deletions of the DFNB1 locus at chromosome 13q11-q12 have been described previously but are uncommon in most populations. This locus includes *GJB2* and *GJB6* encoding connexin 26 and connexin 30, respectively, the two main connexins expressed in the cochlea. To date, four recessive *GJB6* mutations have been reported [25–32]. The two most common are del(*GJB6*-D13S1830) and del(*GJB6*-D13S1854), which truncate *GJB6* and affect expression levels of the *GJB2* gene [33, 34]. The other two are private. One deletes both *GJB2* and *GJB6* [31] and the other (del(chr13:19,837,343-19,968,698) lies upstream from the *GJB6* gene and does not affect either gene directly [30, 32].

In our study, we discovered two probands with heterozygous ~232 kb del(*GJB6*-D13S1854) deletions in the DFNB1 locus encompassing parts of the *GJB6* and *CRYL1* genes and the putative regulatory region of the *GJB2* gene. These probands carry additional heterozygous deleterious point mutations in the *GJB2* gene as discovered by the APEX array and Sanger sequencing; the missense p.Gln80Pro and frameshift g.35delG mutations respectively. Although we cannot determine from our data whether the deletion and point mutations occur in *cis-* or *trans-* configurations, it is likely that the compounded effects of a point mutation in *GJB2* and a deletion of its putative regulatory elements explain the hearing loss in these probands. Our cohort also contained one proband and one control who were carriers of a previously identified heterozygous ~309 kb del(*GJB6*-D13S1830) deletion, which was confirmed by our CNV analysis.

Additionally, we discovered a novel smaller heterozygous deletion (~2-4 kb) in the DFNB1 locus that was called by at least one algorithm in 19 affected individuals and 40 controls. This deletion is ~60 kb upstream of the *GJB6* gene and does not overlap any other genes. Of the 19 cases with this deletion, nine carried an additional known heterozygous deleterious mutation in *GJB2* that was determined by the arrayed primer extension (APEX) array and Sanger sequencing. It is possible that this 2 kb deletion may overlap regulatory elements of the *GJB2* or *GJB6* genes. The combined effects of the deletion and deleterious point mutations may explain the hearing loss in these nine probands. The remaining cases may contain unidentified deleterious mutations on the non-deleted allele while the controls do not. Alternatively, the 2–4 kb deletion may simply be a benign common CNV. With the current data set, we cannot resolve these possibilities.

## Discussion

Using exome sequencing we have identified defects in a myosin II gene, *MYH7B*, as the likely contributors to hearing loss in one family. Although several other myosin heavy chain genes have been previously implicated in hearing loss, the *MYH7B* gene has not. However, there is indirect support for *MYH7B* involvement in hearing including expression in embryonic mouse inner ear tissue [22], expression in the primary auditory complex in humans, and the control of dendritic spine morphology, excitatory synaptic strength, and the actin cytoskeleton in rat neurons [23]. This, along with the segregation pattern, rarity, high quality, and location of the variants in functional domains strongly suggest that the predicted deleterious compound heterozygous mutations in *MYH7B* cause the hearing loss in family 1. The same sequence changes may also be responsible for the megalocornea phenotype in this family as it has been shown that *MYH7B* transcripts are present in extraocular muscles from human, rat, and mouse, and in developing mouse eye skeletal muscle [35, 36]. Changes in extraocular muscle tension can produce significant changes in corneal topography [37]. Interestingly, a single proband in our CNV cohort was found to harbor a deletion of the *MYH7B* gene, as called by Nexus. Our results extend the role of cytoskeletal proteins in hearing and offer the possibility that mutations in the *MYH7B* gene may constitute a rare cause of hearing loss.

Exome sequencing of isolated probands revealed likely causative variants for hearing loss in five cases. Interestingly, two of these probands had two homozygous rare mutations in known hearing loss genes. The rarity of such an occurrence suggests that it is plausible that both mutations may be required for hearing loss. For the remaining probands, multiple homozygous mutations in genes in hearing-relevant pathways and multiple heterozygous deleterious mutations were present. It is possible that hearing loss in these patients is due to rare deleterious homozygous mutations in novel hearing-associated genes, or to codominant, compound heterozygous, or non-allelic non-complementation of heterozygous mutations in distinct genes previously not known to affect hearing. Despite extensive variant filtering and prioritization we are still left with unmanageable numbers of potentially causative mutations in many probands, which we were unable to further refine. Complete distillation of the extensive findings of potentially causative mutations will require expression database analysis (e.g. http://hereditaryhearingloss.org/main.aspx?c=.HHH&n=86597), functional assays in cell and animal models, meta-association analyses of integrated data from multiple genomic studies, and development of novel methods for discerning combinatorial effects of variants.

To our knowledge, this is the first high-resolution genome-wide CNV association study of hearing loss. We discovered novel CNV associations in both known hearing loss-associated genes and in novel candidates. Of note, we found a strong association between a deletion encompassing part of the *PDXDC1* gene and hearing loss. The function of this gene is unknown but it is widely and highly expressed in the cerebral cortex, including the primary auditory cortex, in newborn and adult mice [38] (http://mouse.brain-map.org/experiment/show?id=77869146). This work suggests a need to extend the types of variants typically analyzed in diagnostic hearing loss testing. Furthermore, we have shown the importance of testing for multiple types of variants occurring in combination in individual probands, such as known deletions and point mutations in the *DFNB1* locus. While these heterozygous mutations do not individually explain the phenotype, their compounded effects may well be pathogenic.

Although we were unable to definitively identify the causative SNHL variants for many probands in our cohort, we have found novel mutations that have credible potential to cause or contribute to hearing loss. Maintaining accurate and comprehensive databases will be paramount in driving progress in molecular hearing loss diagnoses.

## Conclusions

Our studies have revealed three important aspects of identifying mutations associated with SNHL. First, exome sequencing of families can reveal novel mutations segregating with SNHL, although not in every instance. Second, exome sequencing of a small number of isolated probands can reveal strong candidate hearing loss mutations, although in some cases it remains challenging to ascertain disease-causing mutations. Third, analysis of CNVs can reveal novel mutations and loci associated with hearing loss. By employing both familial and association studies we have successfully identified rare and potentially private as well as more frequent variants in both novel and previously known candidate genes and loci. Our results indicate that multiple strategies and study designs will be necessary to fully resolve the entire collection of mutations that underlie complex human disorders such as hearing loss. We anticipate that future advances in methods to determine the combinatorial effects of mutations will enable effective assessment of factors including long-range genetic interactions, and will facilitate integrated association analyses of panels of variants and specific phenotypes. At present however, studies like this continue to reveal novel aspects of the multifaceted and expansive genetic architecture underlying hearing loss.

## Methods

### Ethics statement

Informed consent, including consent to publish, was obtained from all enrolled study subjects or their guardians under Internal Review Board approved protocols from Stanford University Medical Center. Controls were recruited under informed consent, including consent to publish, as part of Internal Review Board protocols at Stanford University, Mount Sinai University, and Yale University.

### Sample selection

#### Exome sequencing study samples

The study included 13 probands who were diagnosed with bilateral non-syndromic SNHL, ranging in severity from mild to profound. In addition, the study encompassed parents and siblings of another three probands with SNHL, for a total number of 27 study participants (Table 1). The average age of the probands was four years. Study subjects were enrolled at Stanford University under IRB approval. Prior to inclusion, the probands were, at a minimum, tested for mutations in the *GJB2* gene by DNA sequencing, as part of their routine clinical care. Probands were eligible for this study if this or additional testing had identified no conclusive genetic etiology for their hearing loss. Genomic DNA was isolated from peripheral blood by standard methods. Mutation analysis by APEX microarray identified or confirmed sequence variants in 16 of the 18 probands (data not shown)[39]. Individuals with environmental causes for the hearing loss, which may include a history of trauma, exposure to noise or ototoxic medications, intra-uterine infection, and tumors or other conditions that can affect hearing, were excluded. Individuals with a recognized genetic syndrome were also excluded from this study.

#### CNV study samples

The 150 participating individuals were mostly children; the average age was 10 years. These probands had bilateral non-syndromic sensorineural hearing loss ranging from mild to profound. They were recruited at Stanford University under IRB approval. All probands were tested for mutations in *GJB2* prior to enrollment. Identical selection criteria applied to the different study groups. The set of 13 probands analyzed by whole exome sequencing were included in the CNV analysis.

Genomic DNA was isolated from peripheral blood by standard methods. Mutation analysis by an APEX microarray identified or confirmed sequence variants in 117 of the probands (data not shown) and 44 of these were additionally tested for mutations in the promoter and in exon 1 of the *GJB2* gene (data not shown). Controls for these participants were matched unaffected individuals of the same sex, age range (or older), and in the same ethnic group to the extent possible. Controls were recruited under informed consent as part of IRB protocols at Stanford University (n = 31), Mount Sinai University (n = 88), and Yale University (n = 38) (Table 2).

#### APEX microarrays

The hereditary hearing loss APEX microarray (Asper Biotech, Estonia) contained 198 sequence variants in eight genes (*GJB2*, *GJB6*, *GJB3*, *GJA1*, *SLC26A4*, *SLC26A5*, *MTRNR1*, and *MTTS1*) associated with, mostly, non-syndromic SNHL. These microarrays were used and analyzed as previously described [39].

### Exome sequencing and SNP/indel calling

Exome capture and library preparation was performed using the Agilent SureSelectXT HumanAllExon V4 (50 Mb, product No. 5190–4631). Briefly, 3 μg of gDNA was sheared to a peak size of 150–200 bp using Covaris. Fragmented DNA was cleaned with AmpPure XP beads to remove fragments < 100 bp. The purified DNA fragments were then end-repaired, A-tailed and ligated to indexing-specific paired-end adaptor using the Agilent SureSelect Library Prep Kit, ILM, according to the manufacturer’s instructions.

The adaptor-ligated libraries were amplified for five cycles with the SureSelect Primer and the SureSelect Indexing Pre-Capture reverse primer. PCR reactions were cleaned using the Agencourt AMPure XP. To capture exonic regions, 500 ng of each prepared library was hybridized to biotinylated cRNA oligonucleotides for 24 hours at 65°C. The captured libraries were pulled down using Dynabeads MyOne Streptavidin T1. A post capture PCR was then performed to amplify the captured libraries and to add the barcode sequences for multiplex sequencing for 14 cycles. Amplified libraries were purified with AmpPure XP beads. Qubit fluorometer and Bioanalyzer high sensitivity chips were used to determine the final concentration of each captured library. One library was prepared per sample. Libraries were pooled in pairs, and each pair of libraries was paired-end sequenced on a single Illumina HiSeq lane at the Stanford Center for Genomics and Personalized Medicine according to standard protocols.

Raw fastq files were aligned to hg19, and SNPs and indels were called using two separate pipelines. Fastq files were aligned to the hg19 using DNAnexus mapper with default settings and variants were called using the DNAnexus Nucleotide-level Variation tool. In addition, sequence data from the family pedigrees were aligned to the human reference (hg19 with decoys) and variant identification was performed with the RTG Variant 1.0 software (commercially available from Real Time Genomics, San Bruno, CA). This software includes a read hash-table based alignment step with base recalibration, and a Bayesian variant caller that performs simultaneous multi-sample scoring for pedigrees and uses priors for Mendelian variant segregation ([40]; see Additional file 3 for more details). Sex chromosomes are handled as special cases, and offspring genotypes are phased by transmission.

### Variant filtering

Variants called in the individual probands by DNAnexus were filtered using Ingenuity Variant Analysis as follows. Variants with a call quality of at least 20.0 were kept. Then variants that were observed with an allele frequency ≥ 15.0% of the genomes in the 1000 genomes project (v3), or ≥ 15.0% of the public Complete Genomics genomes (11/2011), or ≥ 15.0% of the NHLBI ESP exomes (All) were excluded. Then variants that were experimentally observed to be associated with a phenotype: Pathogenic, Possibly Pathogenic, Unknown Significance, or established gain of function in the literature, or gene fusions, or inferred activating mutations by Ingenuity, or predicted gain of function by BSIFT, or in a microRNA binding site, or Frameshift, in-frame indel, or stop codon change, or Missense, or disrupt splice site up to 2.0 bases into intron, or deleterious to a microRNA, or structural variant were kept. The Ingenuity Variant Analysis version used was 2.1.20130711. The content versions used were: Ingenuity Knowledge Base (Xiphias _130613.000), COSMIC (v64), dbSNP (Build 137), 1000 Genome Frequency (v3), TargetScan (v6.2), EVS (ESP6500 0.0.19), JASPAR (10/12/2009), PhyloP hg18 (11/2009), PhyloP hg19 (01/2009), Vista Enhancer hg18 (10/27/2007), Vista Enhancer hg19 (12/26/2010), CGI Genomes (11/2011), SIFT (01/2013), BSIFT (01/2013), TCGA (5/14/2012), PolyPhen-2 (HumVar Training set 2011_12), Clinvar (4/8/2013). We also removed variants lying in genes that have emerged as hyper-variable in published exome-sequencing studies in some analyses. The variant filtering scheme for each family and the isolated cases were slightly different. These differences are discussed with the results for each sample set. Of note, for the family analysis a threshold of 3.0% rather than 15.0% frequency in the public genomes in order to study the rarest deleterious mutations segregating in the families.

### Sanger sequencing validation of *MYH7B*variants

Sequences surrounding the two missense mutations (v1 and v2) were amplified by PCR using the Finzymes Phusion High-Fidelity PCR Master Mix (Thermo Scientific) and the following forward (F) and reverse (R) primers:

- v1F - 5’ CGG CTC AAG AAG AAG ATG GA
- v1R - 5’ CCT GCT CGT GGA GCT CAG
- v2F - 5’ GCA GTT CTT CAA CCA GCA CA
- v2R - 5’ ACA CCC TCC CTT CCT CAA AG

PCR cycling was carried out using an optimized version of the manufacturer’s protocol involving 35 cycles with a 30 s annealing step at 65°C and a 10 s elongation step at 72°C. The PCR products were purified using gel electrophoresis followed by extraction using a Qiagen MinElute Gel Extraction kit. The purified products were sent to Elim Biopharmaceuticals (Hayward, CA, U.S.A.) for Sanger sequencing using the following sequencing primers.

- v1 - 5’ ATG GAG GGT GAC CTC AAC GA
- v2 - 5’ TTC CTC AAA GTG ACC TTG CC

The chromatograms were visualized using a demo version of the Sequencher 5.1 (build 10625) software.

### Genome-wide copy number analysis

CNVs were mapped genome-wide to hg18 in all samples by aCGH using the NimbleGen 2.1 M CNV array (Roche NimbleGen) followed by analysis in Nexus Copy Number 6 (Biodiscovery) and NimbleScan 2.6 (Roche NimbleGen). Genomic DNA from each sample was labeled with cy3 dye and genomic DNA from a control pool of seven female individuals (Promega) was labeled using cy5 dye according to the NimbleGen CGH protocol. 34 μg of test and control DNA were mixed together and hybridized to an array for 60–72 hrs. The arrays were washed using the NimbleGen Wash kit and scanned using the MS 200 scanner (Roche NimbleGen) in two channels: 532 nm and 635 nm. Images were normalized using NimbleScan 2.6 (NS). Normalized data were used to derive LRRs using two algorithms: NimbleScan 2.6 segMNT algorithm (default parameters) and Nexus Copy Number 6.0 Rank Segmentation algorithm (significance threshold = 1.0^-9^). Data were loaded into Nexus 6.0 and copy number calls were generated genome-wide for each sample based on fixed thresholds for deletions and duplications specified in the settings.

#### Quality control

Samples were only included in the subsequent analysis if their hybridization passed two quality control filters. The first quality control metric is the *mad1.dr* score calculated by the segMNT algorithm in NimbleScan 2.6. This score is the median absolute deviation of the LRR difference between consecutive probes along the chromosome and is a proxy for the overall noisiness of the hybridization. Hybridizations obtaining a *mad1.dr* score of more than 0.23 are considered by the manufacturer too noisy to be able to discern true differential hybridization from background noise. The second quality control filter, the *Robust Variance Sample QC* score calculated by Nexus 6.0, is also a measure of probe noise. The probe-to-probe variance is calculated but the quality control score takes into account that a certain percentage of variance outliers are expected due to CNV breakpoints. The score is calculated by ordering the magnitudes of the variance between adjacent probes, and then removing the top and bottom 3% of values. The Nexus recommendation for an acceptable *Robust Variance Sample QC* score is less than 0.15-0.2.

### CNV association analysis

#### Genomic region association

 CNV association analysis was carried out using the *Comparisons* function of Nexus 6 with the *classic* option. A Fisher’s Exact Test was performed to determine if the difference between the frequencies of a CNV region in the cases and in the controls is significant. The output of the *Comparisons* function is a list of regions meeting a maximum p-value (*max p-value*) and frequency difference (*differential threshold*) between the case and control groups. These regions are reported in a table such that each region has constant frequency. That is, if a contiguous genomic segment for a given event has different frequencies, the region is split into multiple regions. The Q-bound value corrects for multiple testing by performing a False Discovery Rate correction. Regions containing CNVs that were present at a much larger frequency in the cases versus the controls and incorporating functionally interesting elements were considered top candidates for association. Regions containing CNVs at significant frequencies in the cases and at very low frequencies in the controls were selected for manual examination. Odds ratio (OR) and confidence interval (CI) calculations were carried out using MedCalc for Windows, version 12.7.2 (MedCalc Software, Ostend, Belgium).

#### Gene association

 Single genes were tested for association using a custom built Perl algorithm. The HG18 coordinates of each RefSeq gene were obtained from UCSC genome browser tables. An overlap algorithm was applied to determine which RefSeq IDs including 10 kb up- and downstream overlapped a CNV call from the cohort by at least one base pair. Those RefSeq IDs that did contain overlapping CNVs were subjected to a Fisher’s Exact Test to determine whether it was significantly enriched for overlapping CNVs in the cases versus the controls.

#### Pathway association

 46 genes known to be associated with hearing loss[1] were found to be located within 36 biological pathways in the Kyoto Encyclopedia of Genes and Genomes (KEGG) database. These 36 pathways contain 4,548 RefSeq genes in total. Of these genes, 2,729 were affected by a CNV called in our sample set (i.e. each of these genes had a minimum of 1 bp overlap with a CNV call). Each of these 2,729 genes was tested for association with hearing loss as above. In addition, each of the 36 pathways was also tested for association. In each sample a pathway was counted as being affected by CNVs if at least one of its genes was affected by a CNV. A Fisher’s Exact Test was used to determine whether any of the 36 pathways were more significantly affected by CNVs in the cases than in the controls.

### CNV validation

All cases and 28 controls were genotyped on the Illumina Omni-Quad at Centrillion Biosciences. The data were analyzed for CNVs using the cnvPartition algorithm of the Illumina GenomeStudio software suite and CNVision [41]. These data were visualized in Nexus 6. CNVs of interest were validated by comparison to CNV calls from the SNP genotyping data with acceptable overlap.

### Sample ethnicity determination

Illumina Omni-Quad SNP data were used to determine the ethnicities of the cases and to confirm a subset of the self-reported ethnicities in the medical records of the controls. Specifically, we used the markers on the Illumina Human Omni1Quad array that belonged to the Human Genome Diversity Project SNP collection as input. Sample data were formatted using PLINK (http://pngu.mgh.harvard.edu/purcell/plink/) [42]. Principle component analysis was performed to determine the ethnicities of the samples using EIGENSTRAT [43].

### Integrated analysis of CNV and SNV data

Genome-wide CNV and SNV data were overlaid using custom algorithm and IVA in order to detect genomic loci harboring multiple types of deleterious variants in 13 probands. The CNV data were mapped from hg18 to hg19 using the UCSC liftover tool in order to match the SNV data.

## Supplemental Data

### Supplementary methods

#### Real time genomics analysis

Reads were aligned with the RTG map algorithm to the hg19 reference with decoys used by the 1000 Genomes Project^1^. RTG map creates a hash table that indexes the reads and streams the reference sequence to identify mapping locations. Mapping of paired-end reads is performed concurrently in a collection window that is much larger than the library insert size (in this case the window was 1,000 bp). RTG maps also calculate base QV recalibration tables, which are needed for variant calling, and outputs standard BAM format files. The RTG variant caller uses a Bayesian framework (originally proposed by Marth *et al.*^2^) that estimates diploid genotype posterior probabilities per and uses priors for polymorphism rates based on the data of the 1000 Genomes Project^1^. Platform-specific error rates are modelled as priors and mapping quality values from the mapper are incorporated as part of the data. Depth of coverage is also considered during scoring penalizing variants with higher-than expected coverage. For this, depth of coverage needs to be estimated before variant calling; in the case of exomes a BED file with the target regions is used to estimate target depth appropriately. Complex regions are identified by various criteria, mainly including regions with apparent indels, MNPs, or clusters of SNVs. A specialized Bayesian caller is used for these regions (“complex caller”) which iteratively selects pre-existing single-read alignments in the region as hypothesis, aligns the rest of the reads to the hypothesis by a probabilistic Goth algorithm and estimates the posterior probability of each hypothesis considering diploid indels and MNP variants. The final call is the hypothesis with the highest posterior probability and accounts for about 10% of the total variant calls^3^. In the case of data from pedigrees, alignments are evaluated simultaneously across pedigree members at every position using a scoring method that assumes Mendelian variant segregation. Sex chromosomes are handed as special cases. This dramatically reduces Mendelian inconsistencies without filtering of variants, and improves the genotype qualities (GQ) of true positives, while decreasing the GQ of probably false positives (unpublished). In order to evaluate the possibility of *de novo* mutations, a small prior is allowed for such type of events and a specific score is calculated for the *de novo* mutation hypothesis and it is included in the output VCF. In the case of nuclear families, offspring genotypes are phased by transmission. The output is a multi-sample VCF conforming to v 4.1 specifications and includes all variants through the score range (i.e. no filtering is performed by default).

1. Consortium, T. 1. G. P. *et al.* An integrated map of genetic variation from 1,092 human genomes. *Nature***490,** 56–65 (2013).
2. Marth, G. T. *et al.* A general approach to single-nucleotide polymorphism discovery. *Nat. Genet.***23,** 452–456 (1999).
3. Reumers, J. *et al.* Optimized filtering reduces the error rate in detecting genomic variants by short-read sequencing. *Nature Biotechnology***30,** 61–68 (2011).

## Availability of supporting data

The data sets supporting the results of this article are available in the Sequence Read Archive (exome sequencing data) and the Gene Expression Omnibus (microarray data) repositories, http://www.ncbi.nlm.nih.gov/sra (accession number: SRP050895) and http://www.ncbi.nlm.nih.gov/geo/ (accession number: GSE64088).

## Electronic supplementary material

Additional file 1: Figure S1: Distributions of total number of CNV calls per sample. The total number of CNV calls per sample produced by the Nexus (3 leftmost plots) and the NimbleScan (three rightmost plots) algorithms are shown. In addition to the total distributions (yellow), the case (red) and control (green) distributions are shown separately. (TIFF 247 KB)

Additional file 2: Figure S2: Size distributions of CNV calls. The sizes of individual CNV calls produced by the Nexus and NimbleScan algorithms are shown for the case and control sample sets. The apparent frequency spikes are partly the result of changing bin size. (TIFF 2 MB)

Additional file 3: **Supplementary methods describing Real Time Genomics analysis.** (DOCX 12 KB)
