# Supplementary figures and images for: Exome sequencing and genome-wide copy number variant mapping reveal novel associations with sensorineural hereditary hearing loss
Source: BMC Genomics. 2014 Dec 20;15(1):1155. doi: 10.1186/1471-2164-15-1155 (PMC4367882; doi:10.1186/1471-2164-15-1155)

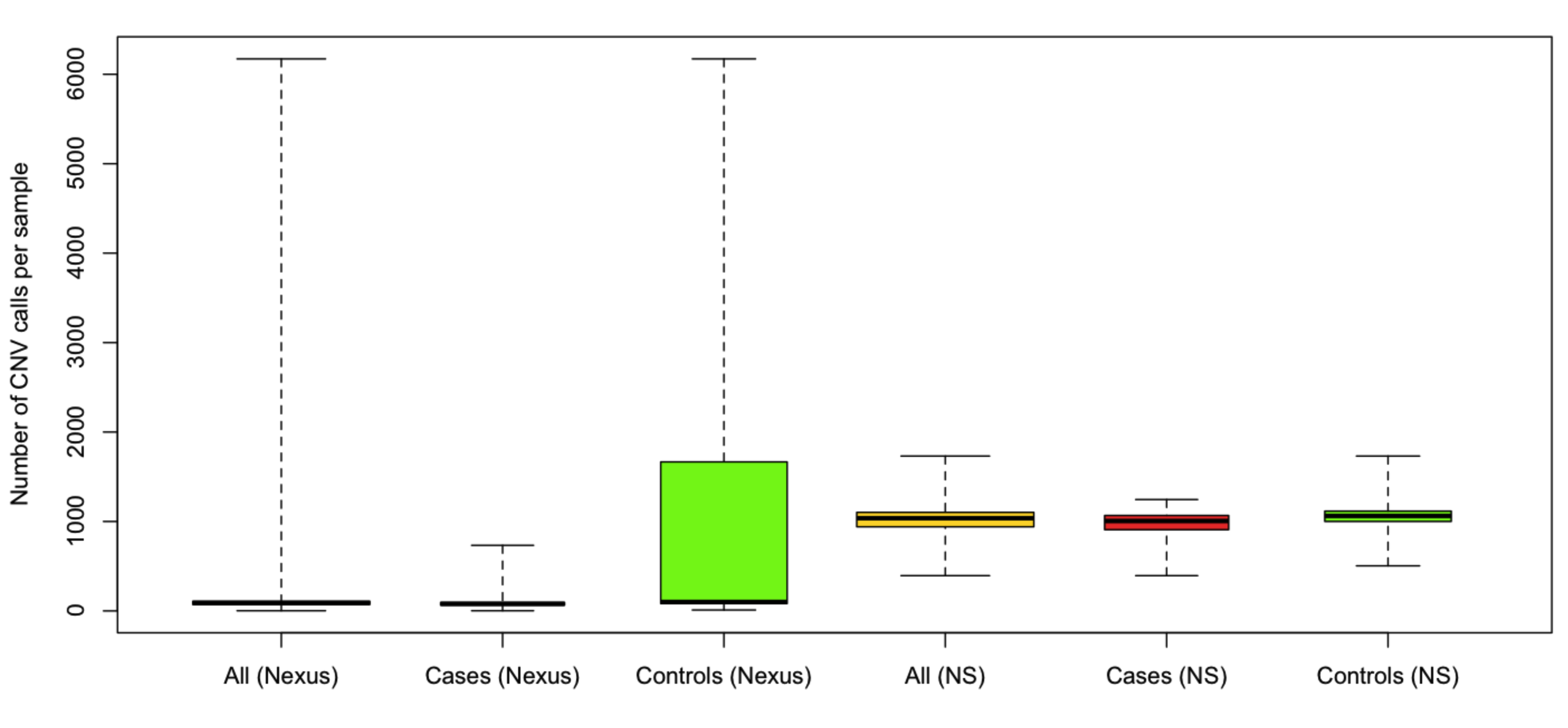

Supplement: Supplementary file 1 — Additional file 1: Figure S1: Distributions of total number of CNV calls per sample. The total number of CNV calls per sample produced by the Nexus (3 leftmost plots) and the NimbleScan (three rightmost plots) algorithms are shown. In addition to the total distributions (yellow), the case (red) and control (green) distributions are shown separately. (TIFF 247 KB) [file 12864_2014_6949_MOESM1_ESM.tiff]

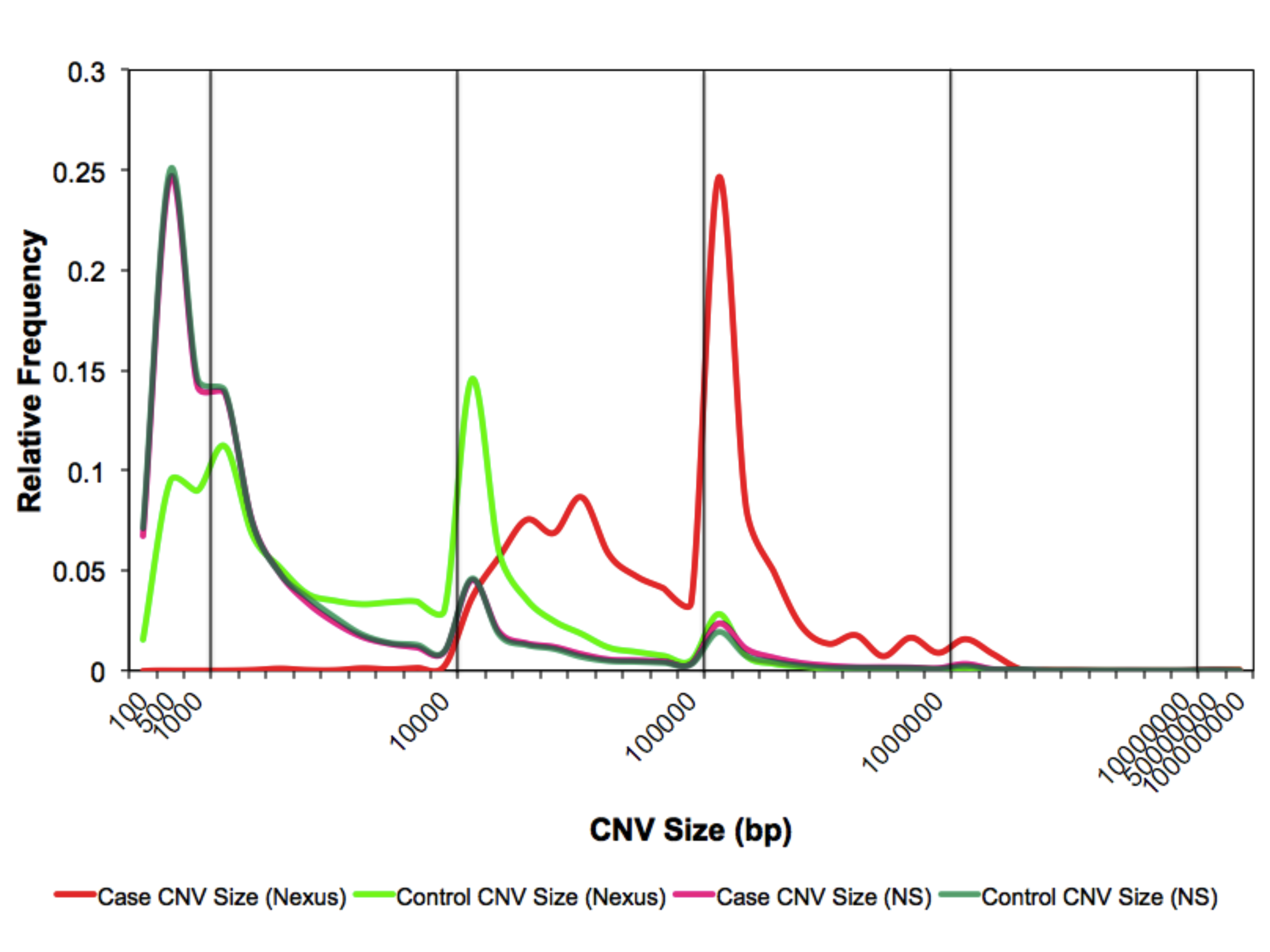

Supplement: Supplementary file 2 — Additional file 2: Figure S2: Size distributions of CNV calls. The sizes of individual CNV calls produced by the Nexus and NimbleScan algorithms are shown for the case and control sample sets. The apparent frequency spikes are partly the result of changing bin size. (TIFF 2 MB) [file 12864_2014_6949_MOESM2_ESM.tiff]
