# Supplementary material for: Exome sequencing and genome-wide copy number variant mapping reveal novel associations with sensorineural hereditary hearing loss
Source: BMC Genomics. 2014 Dec 20;15(1):1155. doi: 10.1186/1471-2164-15-1155 (PMC4367882; doi:10.1186/1471-2164-15-1155)
Supplement: Supplementary file 3 — Additional file 3: Supplementary methods describing Real Time Genomics analysis. (DOCX 12 KB) [file 12864_2014_6949_MOESM3_ESM.docx]

**Additional file 3**

**Supplementary Methods**

Real Time Genomics Analysis

Reads were aligned with the RTG map algorithm to the hg19 reference with decoys used by the 1000 Genomes Project^1^. RTG map creates a hash table that indexes the reads and streams the reference sequence to identify mapping locations. Mapping of paired-end reads is performed concurrently in a collection window that is much larger than the library insert size (in this case the window was 1,000 bp). RTG maps also calculate base QV recalibration tables, which are needed for variant calling, and outputs standard BAM format files. The RTG variant caller uses a Bayesian framework (originally proposed by Marth *et al.*^2^) that estimates diploid genotype posterior probabilities per and uses priors for polymorphism rates based on the data of the 1000 Genomes Project^1^. Platform-specific error rates are modelled as priors and mapping quality values from the mapper are incorporated as part of the data. Depth of coverage is also considered during scoring penalizing variants with higher-than expected coverage. For this, depth of coverage needs to be estimated before variant calling; in the case of exomes a BED file with the target regions is used to estimate target depth appropriately. Complex regions are identified by various criteria, mainly including regions with apparent indels, MNPs, or clusters of SNVs. A specialized Bayesian caller is used for these regions (“complex caller”) which iteratively selects pre-existing single-read alignments in the region as hypothesis, aligns the rest of the reads to the hypothesis by a probabilistic Goth algorithm and estimates the posterior probability of each hypothesis considering diploid indels and MNP variants. The final call is the hypothesis with the highest posterior probability and accounts for about 10% of the total variant calls^3^. In the case of data from pedigrees, alignments are evaluated simultaneously across pedigree members at every position using a scoring method that assumes Mendelian variant segregation. Sex chromosomes are handed as special cases. This dramatically reduces Mendelian inconsistencies without filtering of variants, and improves the genotype qualities (GQ) of true positives, while decreasing the GQ of probably false positives (unpublished). In order to evaluate the possibility of *de novo* mutations, a small prior is allowed for such type of events and a specific score is calculated for the *de novo* mutation hypothesis and it is included in the output VCF. In the case of nuclear families, offspring genotypes are phased by transmission. The output is a multi-sample VCF conforming to v 4.1 specifications and includes all variants through the score range (i.e. no filtering is performed by default).

1. Consortium, T. 1. G. P. *et al.* An integrated map of genetic variation from 1,092 human genomes. *Nature* **490,** 56–65 (2013).

2. Marth, G. T. *et al.* A general approach to single-nucleotide polymorphism discovery. *Nat. Genet.* **23,** 452–456 (1999).

3. Reumers, J. *et al.* Optimized filtering reduces the error rate in detecting genomic variants by short-read sequencing. *Nature Biotechnology* **30,** 61–68 (2011).
